# To the Editors of the Medical and Physical Journal

**Published:** 1806-01

**Authors:** G. P. Dawson


### *To the Editors of the Medical and Physical Journal.*

GENTLEMEN,

PERMIT me to communicate a case of *incipient* Procidentia Uteri, in which the Sponge Pessary was eminently serviceable. The protrusion, by occasioning violent irritation, profuse discharge, and extensive excoriation, was peculiarly distressing to the patient. Ignorant of its true nature, she requested my advice. By suitable inquiries I ascertained the fact, and recommended a pessary of sponge. Its success was adequate to my expectations, and equal to her warmest wishes. The lady, whose case is narrated in No. 72 of your Journal, continues to use the sponge pessary, with unparalleled efficacy. After she had persevered in its use for six months, she, unknown to me, discontinued it, under the idea of being perfectly well, and, notwithstanding this imprudence, the tumour was nearly a month in reappearing. This circumstance strongly indicates the hope of a permanent cure, by unremittingly prosecuting the use of the pessary for a considerable time. For, as it had the effect I have stated, in a case of the worst kind, accompanied by a lacerated perinæum, surely it is not chimerical to expect a radical cure in a recent and *incipient* procidentia uteri, existing in a youthful female. I am thus minute in stating, and anxious in collecting, facts relative to procidentia uteri, from a thorough conviction of its prevalence: assuredly, it is a most unpleasant and harrassing complaint; unquestionably, attempting to relieve it, is no mean occupation. I named the former case procidentia; but, according to the distinctions laid down by writers, it ought to have been prolapsus. I did this designedly. I am averse to the adoption of a multiplicity of technical terms to designate the different stages of this complaint. I think it would be better, certainly more simple, to reject prolapsus, and every other term, and substitute in their stead procidentia. This might readily be accomplished by dividing procidentia uteri into two stages, the *incipient*, and the *complete*; the *incipient* or when the uterus remains within the labia; the *complete* or when it protrudes externally. Your correspondent, Mr. Simpson, has with becoming liberality* subjected the

* I wish every Medical Man possessed this amiable quality. It is a melancholy, but a true reflection, that much illiberality prevails among country practitioners. Let me not be misunderstood. There are hundreds sponge pessary to trial, for which he will accept my thanks, and is pleased to express himself amply convinced of its utility; I can assure this Gentleman it will not disgrace his sanguine recommendation, or discredit the very liberal and handsome encomiums bestowed on its value.

I am, &c.

*Sunderland, Nov.* 5, 1805. G. P. DAWSON.

---

### *To the Editors of the Medical and Physical Journal.*

dreds, some within the sphere of my own acquaintance, of exemplary candour and liberality, an honour to the profession, and to human nature; but there are others, and to them I allude, who are, what a noble author calls, children of a larger growth. These men view their contemporaries with a jaundiced and a jealous eye, and, Zanga like, rapturously embrace every opportunity of doing them disservice. The justly celebrated Dr. Trotter, of Newcastle, a man whose name science cannot but revere, elegantly expresses my sentiments, when he emphatically remarks, "It does not much redound to the honour of the age, that there are men among us, who, assuming all the airs of a Radcliffe, without the talents, are insidious and jealous inquisitors of the practice of others, and surly dictators in their own; whose will is their law: Stat pro ratione voluntas, and "Bear, like the Turk, no brother near the throne." It is, however, exceedingly gratifying to a generous mind, to know that "no divinity hedges" the illiberal and the ungentlemanly. When my avocations will permit, and circumstances will allow, I purpose writing an Essay on Medical Illiberality. A friend of mine, a man of genius and of learning, to whom I imparted my design, observed that such a work would expose venality and ignorance, bestow on modest merit its just reward, punish unblushing effrontery and unworthy arts by deserved contempt, and render futile all the whispers levelled at a neighbour's fame! Could I persuade myself of this, I should not long delay the grateful task.

